# Potential of CoMn_2_O_4_ spinel as soot oxidation catalyst and its kinetics thereof

**DOI:** 10.1038/s41598-025-85736-2

**Published:** 2025-01-07

**Authors:** R. Nithya, Sunaina S. Patil, Hari Prasad Dasari, Harshini Dasari, S. Nethaji

**Affiliations:** 1 Department of Chemical Engineering, Manipal Institute of Technology, Manipal Academy of Higher Education, 576104 Manipal, Karnataka India; 2 Energy & Catalysis Materials Laboratory, Department of Chemical Engineering, National Institute of Technology Karnataka, 575025 Surathkal, Mangalore India

**Keywords:** Reverse co-precipitation, Spinel oxide, Soot oxidation, Soot oxidation kinetics, Chemistry, Energy science and technology, Materials science

## Abstract

Efficient catalysts for soot oxidation are critical for mitigating environmental pollution. In this study, CoMn_2_O_4_ spinel catalysts were synthesised using reverse co-precipitation and co-precipitation methods to evaluate their performance in soot oxidation and kinetic behaviour. All samples exhibited a tetragonal phase (XRD) and spherical morphology with rough surfaces (SEM). Raman spectroscopy confirmed structural disorder and oxygen vacancies, while XPS analysis revealed the presence of low-valence Mn ions, facilitating oxygen vacancy formation critical for soot oxidation. Additionally, the co-existence of Co and Mn ions contributed to a synergistic effect, enhancing the catalytic properties of the spinel structure. The reverse co-precipitation method produced a catalyst with a higher concentration of oxygen vacancies and active oxygen species among the samples. This sample demonstrated superior catalytic performance, achieving a T_50%_ of 424 °C, low activation energy (153 kJ/mol) and pre-exponential factor (25 min^− 1^). Soot TPR analysis highlighted the role of catalyst reducibility, while thermogravimetric analysis revealed that activation energy and pre-exponential factors were influenced by surface composition. These findings provide valuable insights into the design of efficient catalysts for soot oxidation, emphasising the importance of synthesis methods and surface characteristics.

**Supplementary Information:**

The online version contains supplementary material available at 10.1038/s41598-025-85736-2.

## Introduction

Soot particles are formed during incomplete fuel combustion in a diesel engine despite their efficiency and power. The soot particles are a major concern globally as they pose a greater threat to humans and the environment^1,2^. To mitigate soot being released into the environment, diesel filters are employed in exhaust systems. These filters effectively remove the soot particles; however, the soot particles accumulate, causing increased backpressure and degrading the engine performance and fuel efficiency^3^. Therefore, regeneration of DPFs is necessary at regular intervals to oxidise and remove the accumulated soot whilst maintaining the efficiency and durability of the filter^4^.

The regeneration of diesel filters can be achieved through two methods: passive and active regeneration methods. The passive regeneration method involves employing a diesel oxidation catalyst on diesel filters, which reduces the activation energy of soot oxidation, essentially decreasing the soot oxidation temperature^5^. The soot is oxidised continuously at the exhaust working temperature and is converted into less harmful gases such as carbon dioxide and water vapour^6^. This method primarily depends on the catalytic properties of materials such as palladium, platinum, and other noble metals. In contrast, the active regeneration method involves increasing the exhaust temperature through techniques like post-injection fuel or electrical heating, which ensures that the temperature is sufficiently high enough to oxidise the accumulated soot. Although this method is effective, active regeneration requires more fuel and could complicate the engine control system^7–9^.

In the past decades, researchers have explored various catalysts for soot oxidation, such as noble metals, ceria-based, perovskite-based, and transition metal oxides^10^. Among them, spinel oxides are particularly interesting due to their unique properties, such as unique structure, thermal stability, and outstanding catalytic activity, especially in oxidation-type reactions^11–13^. The main advantage of spinel oxides is that the required catalytic property can be tuned by the positions of metal ions at the A and B sites of the spinel structure. Spinel oxides possess several advantages, such as a high number of active sites, strong oxygen binding ability, and mixed valence states that facilitate charge transfer through redox reactions and surface engineering for specific applications. Li et al.,^14^ achieved a high number of active sites by tailoring manganese cobalt spinel oxide for oxygen reduction reaction. Dong et al.,^15^ synthesised CoMn_2_O_4_ with a large surface area, oxygen vacancy and high mobility of active oxygen species for toluene oxidation.

It is evident that the various preparation methods significantly influence the intrinsic properties of catalysts, such as surface area, particle size, and particle distribution.^16,17^ These changes can either enhance or degrade the performance of the catalysts in various applications. Mira et al. studied the effect of different preparation methods on CeZrNd mixed oxide catalyst for soot combustion. The catalyst prepared by the microemulsion method had the highest catalytic activity compared with the co-precipitation method^18^. Díaz et al.,^19^ studied the effect of different synthesis methods on soot oxidation and concluded that the synthesis methods significantly impacted the catalyst’s properties and activity. According to Díaz, the most active catalyst for soot oxidation is synthesised via the sol-gel (Sg) method (T_50%_=435 °C), followed by microwave (MW) and self-combustion (SC) methods. Although the Sg-synthesised material excelled in activity, the MW-synthesized material demonstrated superior thermal stability and selectivity towards CO_2_ after six cycles, making it a promising candidate for long-term applications.

Understanding the kinetics of Diesel Particulate Matter (DPM) oxidation is crucial for designing effective DPFs. Kinetic analysis helps study the oxidation of carbonaceous PM to understand soot-burning behaviour. Soot oxidation is a complex heterogeneous reaction, differing from homogeneous reactions, and its reactivity varies within the solid particle. Both single-step and multi-step reaction mechanisms are used to explain the combustion processes. The kinetic analysis of solid-state decompositions is typically based on a single-step kinetic equation involving time, temperature, extent of conversion, and the reaction model. Thermogravimetric analysis (TGA) is extensively used to study the oxidation kinetics of diesel exhaust soot. Yet, it does not directly provide the kinetic triplet (activation energy, pre-exponential factor, and reaction model). Traditional model-fitting methods yield a single activation energy value, disregarding its variation with the degree of conversion.

Conversely, model-free methods reveal that activation energy changes with conversion, indicating a multi-step oxidation process. For accurate kinetic analysis, it is crucial to consider both activation energy and the pre-exponential factor, as the reaction rate depends on both. Including these factors offers a more comprehensive understanding of the soot oxidation process.

This work studied the effect of the synthesis method of CoMn_2_O_4_ spinel oxide on soot oxidation. Additionally, the kinetic triplet of the soot oxidation reaction was investigated in detail based on non-isothermal thermogravimetric data. The model-free method was used to estimate variations in activation energy during oxidation, by which the reaction steps were determined.

## Experimental section

### Catalyst Preparation

#### Reverse co-precipitation

An appropriate amount of Cobalt Nitrate and Manganese Nitrate was taken in a separate beaker containing distilled water. The solution was transferred into two separate burettes and set to drop simultaneously into a beaker containing ammonia solution. After completing the dropdown from both burettes, the mixture was stirred for two hours. The precipitate obtained was washed and dried at 80 °C and calcined at 650 °C. The prepared catalyst was named RCOP_CoMn_2_O_4_^20^.

#### Co-precipitation

An appropriate amount of Cobalt Nitrate was taken in a beaker containing distilled water and stirred for a few minutes to attain a homogeneous solution. A proper amount of ammonia solution was dropped down to the obtained solution. Manganese nitrate solution was added dropwise into the mixture and was stirred for two hours. The precipitate obtained was washed and dried at 80 °C and calcined at 650 °C. The prepared catalyst was named COP_C_ CoMn_2_O_4_. To prepare the COP_M_CoMn_2_O_4_ sample, manganese nitrate was dropped into the ammonia solution instead of first adding cobalt nitrate into the ammonia solution. The rest of the procedure followed the same method as above^20^.

### Characterisation techniques

The powder X-ray diffraction method examined the sample structure and phase purity. At a scanning rate of 0.02 °s^− 1^, the X-ray source of λ = 1.54Å was employed in step scan mode in the 2θ range from 10° to 80°. By using the Scherrer Equation, the average crystallite size was calculated. The morphology of the prepared samples was imaged using a Scanning Electron Microscope (SEM) (EVO MA18 with Oxford EDS(X-act)). This method facilitated the identification of active oxygen species present on the catalyst surface. An excitation wavelength of 785 nm was used to record the Raman spectra using a Compact Raman Spectrometer, Renishaw (UK). X-ray photoelectron spectroscopy (XPS) analysis was conducted using a SPECS instrument from Germany, employing AlKα radiation for excitation. The binding energies were calibrated relative to C 1s at 284.6 electron volts (eV). At a gas flow rate of 60 mL/min, soot TPR was performed in the presence of a nitrogen atmosphere, and the mixed sample was fed into the instrument with a heating temperature range of 50 to 800 °C. It helps to identify active oxygen species on the surface of catalysts.

### Catalytic activity and kinetic analysis

The catalytic soot oxidation was analysed using thermogravimetric analyses. The catalyst and soot were combined in a ratio of 10:1, and around 15 mg of the mixed sample was fed into the instrument. It was performed using a thermogravimetric analyser (a Hi-Res TGA 2950) with 60 mL/min at a heating rate of 10 °C/min from room temperature to 700 °C.

To determine the kinetic triplets—pre-exponential factor (A), activation energy (Ea), and reaction model—solid-state reaction kinetics analysis is carried out. The TGA measured the soot oxidation kinetics at various heating rates: 5, 10, 15, and 20 °C/min. The KAS approach and the Flyn Wall Ozawa method were utilised to calculate activation energy (Ea). The Coats-Redfern approach is one of the most often used non-isothermal model fitting techniques for Arrhenius factor and activation energy determination. The pre-exponential factor was determined using the Avrami method. The optimal reaction model for solid-gas reactions is identified by comparing computed and experimental values on a master plot.

## Results and discussion

### Catalyst Characterisation

**Fig. 1:**
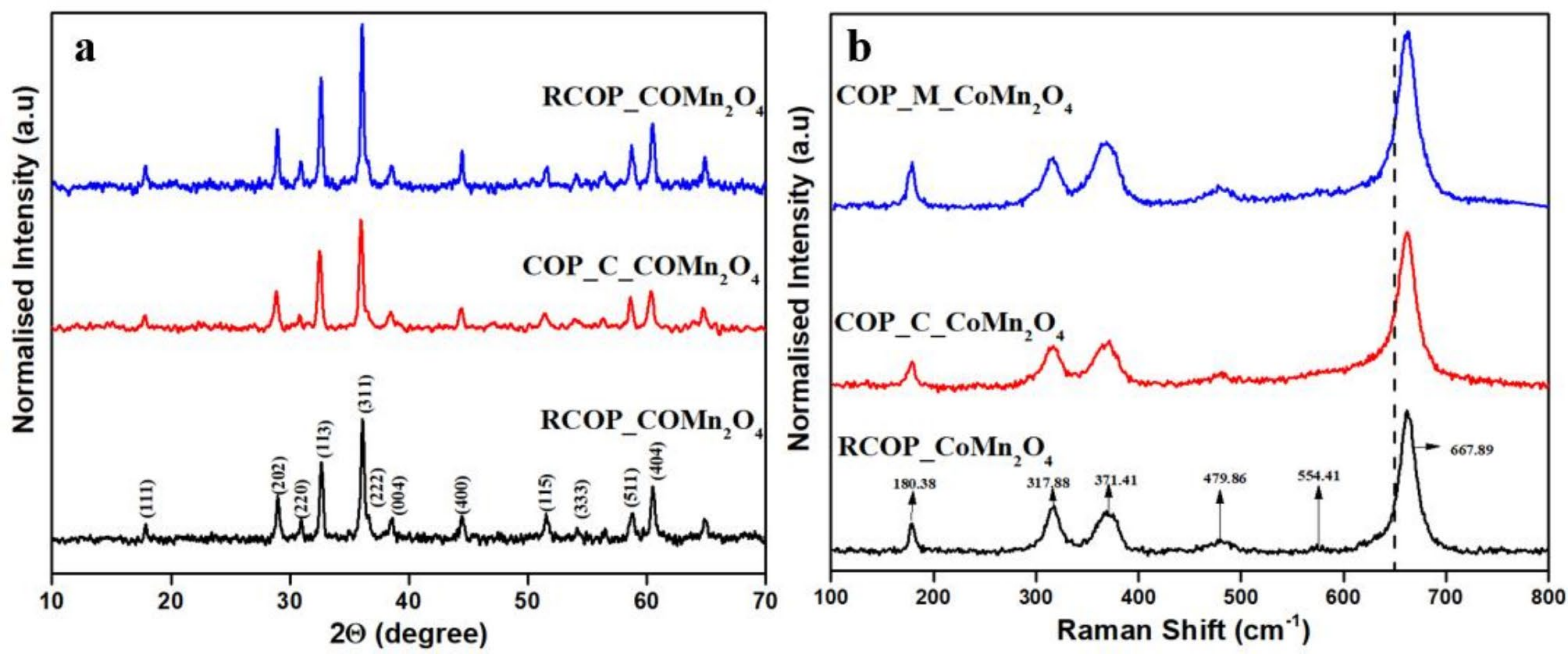
(**a**) XRD spectra of the samples RCOP_ CoMn_2_O_4_, COP_C_ CoMn_2_O_4_, and COP_M_ CoMn_2_O_4_ (**b**) Raman Spectra of the samples RCOP_ CoMn_2_O_4_, COP_C_ CoMn_2_O_4_, and COP_M_ CoMn_2_O_4_.

The catalysts synthesised from reverse co-precipitation and co-precipitation exhibited a tetragonal crystal structure, as confirmed by X-ray diffraction (XRD) analysis (Fig. 1a). The diffraction patterns displayed characteristic peaks at 2θ values of 18.16°, 29.20°, 31.13°, 32.85°, 36.34°, 38.77°, 44.63°, 52.63°, 54.22°, 56.62°, 59.03°, 60.63°, and 65.24°. These peaks correspond to the standard reference data for CoMn_2_O_4_ (ICDD No: 00-055-0685) and align with the reported literature^21^. The absence of additional peaks in the XRD spectra shows the purity of synthesised samples. Additionally, there was no visible shift in the XRD peaks for the synthesised samples, implying that the preparation methods did not induce any significant changes in the crystal structure. The narrow and sharp peaks indicate the good crystalline structure of the synthesised materials^22^. Table S1 provides the average crystallite size, lattice parameters, and BET surface area of the synthesised catalysts.

The Raman spectra analysis synthesised CoMn_2_O_4_ samples, as shown in Fig. 1b, peaks and vibrational modes, per previous literature. The distinct peak around 180.38 cm ^− 1^ is associated with the vibrational modes of CoO_4_ at the tetrahedral site and CoO_6_ at the octahedral site, thus showing cobalt-oxygen bonding in different coordination environments. Peaks around 317.88 cm^− 1^ and 371.41 cm^− 1^ are attributed to oxygen movement within MnO_6_ and MnO_4_ sites, indicating the dynamic nature of oxygen atoms in manganese coordination environments^23^. The peak at 479.86 cm^− 1^ relates to vibrations of cations at the tetrahedral sites, while the peak at 554.41 cm^− 1^ is ascribed to crystal lattice oxygen vibrations^24,25^. The peak at 554.41 cm^− 1^ is noticed for RCOP_CoMn_2_O_4_ but not in the other two samples. It signifies the presence of lattice oxygen in the crystal structure of RCOP_CoMn_2_O_4_.

Furthermore, the oxygen atoms with vibrational mode in an octahedral site have a characteristic peak, usually about 650 cm^− 1^^23^. The peak positions are consistent with the reported literature^26^. However, the blue shift for all samples indicates the presence of oxygen vacancies that have disturbed the local environment and vibrational frequencies of oxygen atoms. It collectively suggests the presence of mixed valence states of cobalt and manganese within the CoMn_2_O_4_ structure^27,28^. Similarly, Pathak et al.^29^ observed a blue shift in the peaks of NiCo_2_O_4,_ which confirmed the presence of oxygen vacancies. The blue shift positively enhances the catalytic activity by providing additional active sites and facilitating oxygen mobility^24^. In conclusion, the Raman spectra confirm the characteristic vibrational modes of CoMn_2_O_4_ while emphasising the influence of oxygen vacancies on the material’s structural and catalytic properties, underscoring the complex interplay of factors contributing to its performance in various applications.

**Fig. 2:**
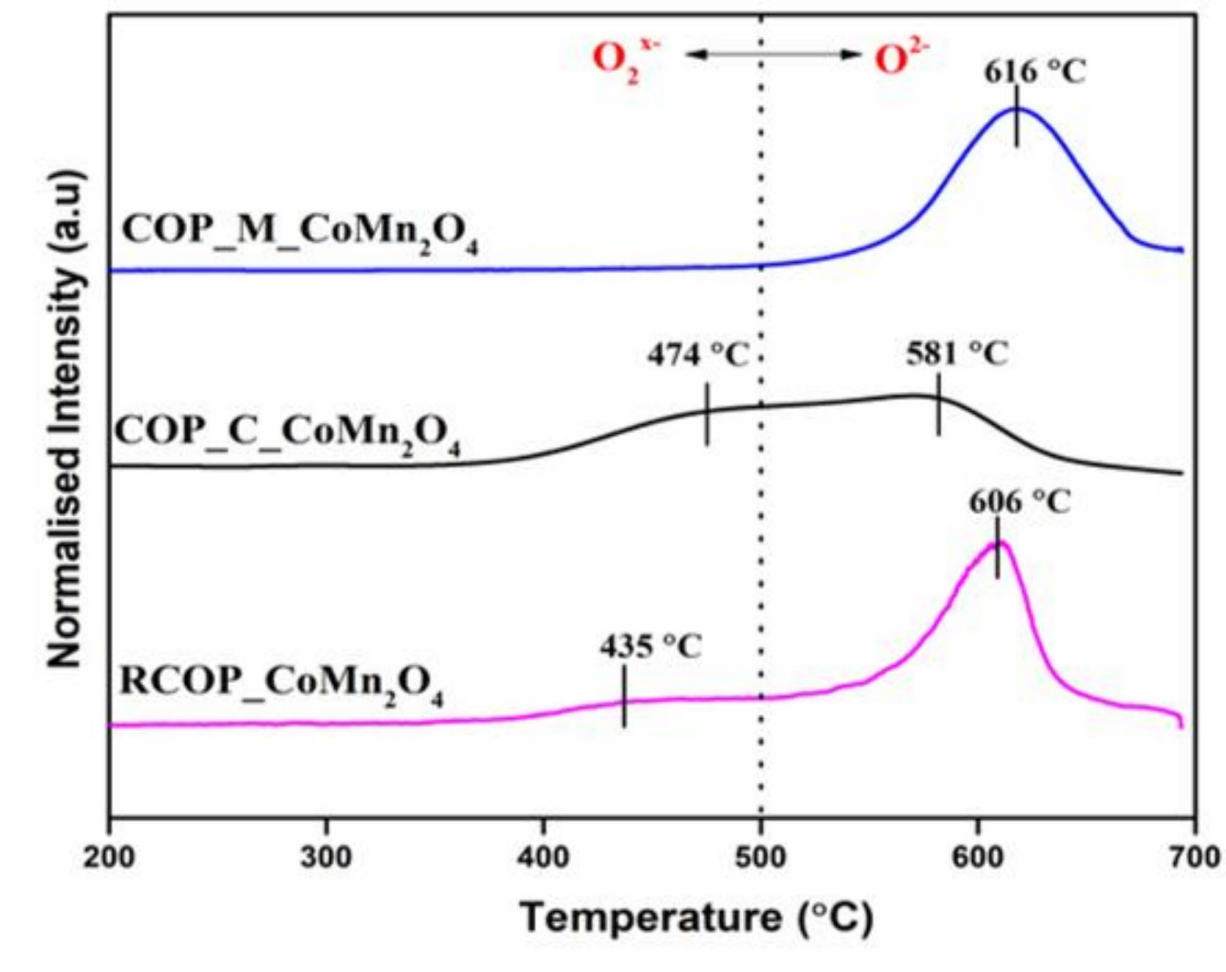
Soot TPR analysis under inert atmosphere on samples RCOP_ CoMn_2_O_4_, COP_C_ CoMn_2_O_4_, and COP_M_ CoMn_2_O_4_.

The soot TPR analysis (Fig. 2) shows the catalyst’s capability to oxidise soot in an inert atmosphere by utilising the active oxygen species on the catalyst surface. The redox property of the catalyst also plays an essential role in generating active oxygen species to oxide soot. Two types of oxygen species are present on the catalyst surface according to their reducibility propensity. (a) Surface adsorbed oxygen species (O_2_^−^) released at lower temperatures (200–500 °C). (b) Lattice oxygen species (O^2−^) are only emitted at high temperatures (more than 500 °C). In nature, O_2_^−^ species are loosely attached to the surface and highly active, whereas O^2−^ species are difficult to release from the catalyst^30^. Both types of oxygen species are present in the RCOP_CoMn_2_O_4_ and COP_C_ CoMn_2_O_4_ samples. Since surface-adsorbed oxygen species have a substantial reactive property, they quickly oxidise soot particles at low temperatures. Besides, the area of the curve under the peak 443 °C for the RCOP_CoMn_2_O_4_ sample is less compared with the area under the peak 474 °C, demonstrating less surface adsorbed oxygen species. However, the surface-adsorbed oxygen species are quickly released at lower temperatures, which implies that RCOP_ CoMn_2_O_4_ may exhibit lower soot oxidisation temperature. Thus, we can say that the COP_C_ CoMn_2_O_4_ samples may oxidise soot at lower temperatures than RCOP_ CoMn_2_O_4_ samples. Additionally, COP_C_ CoMn_2_O_4_ samples can release lattice oxygen species at 581 °C, a much lower temperature than RCOP_CoMn_2_O_4_ samples (606 °C). The reason may be that the reducibility property of COP_C_ CoMn_2_O_4_ samples is higher than that of the RCOP_ CoMn_2_O_4_ sample. Moreover, the intermediate formation during the synthesis of the COP_C_ CoMn_2_O_4_ sample may have increased the number of oxygen vacancies in the catalyst^31^. The presence of only lattice oxygen species in COP_M_ CoMn_2_O_4_ samples may affect the performance of soot oxidation activity. It may oxidise soot at higher temperatures as the release of lattice oxygen species takes place at higher temperatures, and it also depends upon the reducibility of the catalyst.

**Fig. 3:**
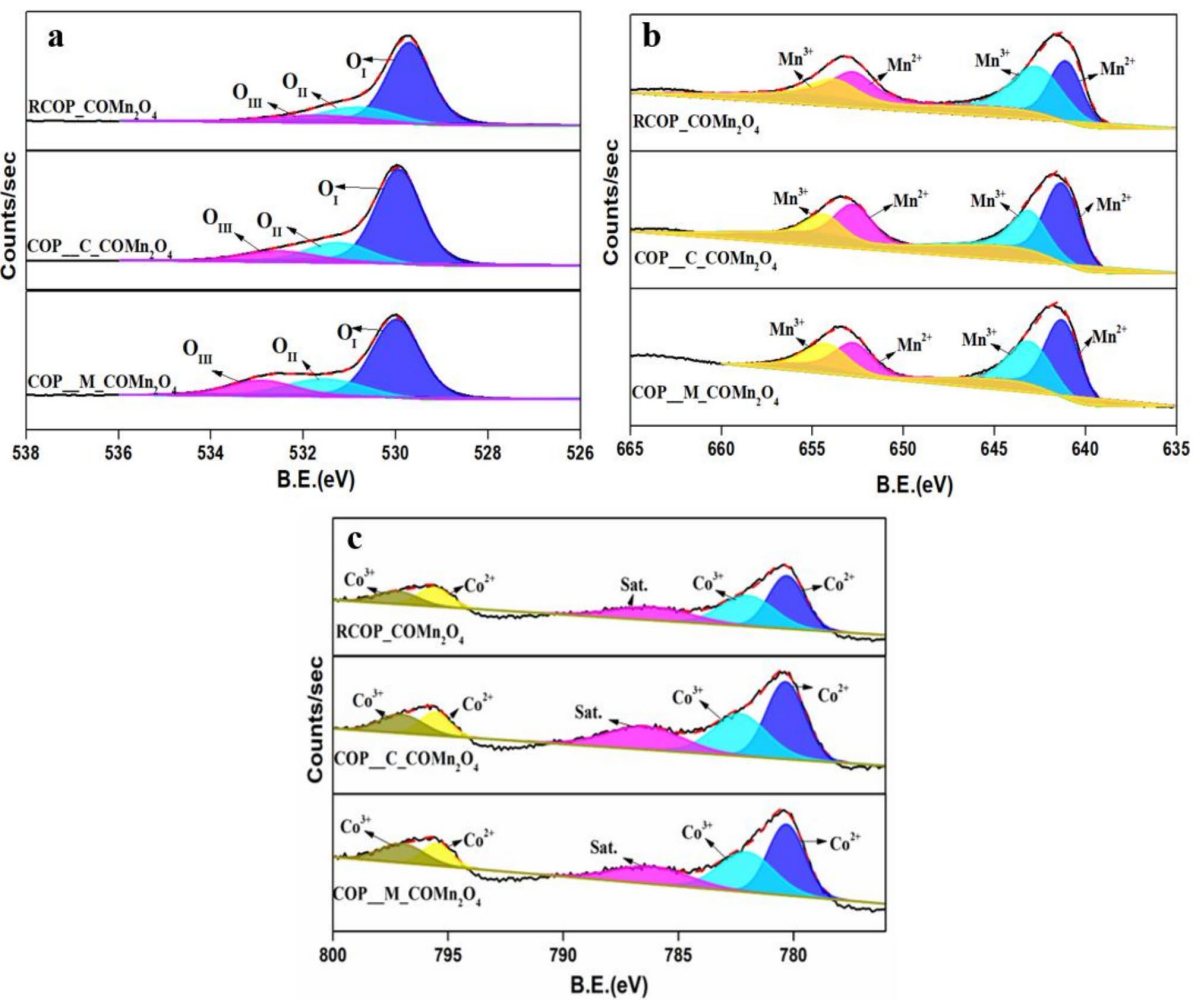
XPS analysis of (**a**) O 1s (**b**) Mn 2p (**c**) Co 2p of the samples RCOP_ CoMn_2_O_4_, COP_C_ CoMn_2_O_4_, and COP_M_ CoMn_2_O_4_.

XPS analysis provides detailed insights into the defects and oxidation states of the prepared CoMn_2_O_4_ samples. Figure 3a, b and c depict the XPS spectra for the O1s, Mn 2p, and Co 2p core levels for all the synthesised samples. The Mn 2p peaks of all synthesised samples centred at 653.9 eV and 642.9 eV ascribe to the Mn 2p_1/2_ and Mn 2p_3/2_ of the Mn^3+^ ion, respectively. The peaks centred at 652.4 eV and 641.3 eV are ascribed to the Mn 2p_1/2_ and Mn 2p_3/2_ of the Mn^2+^ ion, respectively. All the peaks are well in accord with the literature^32^. The peaks affirm the presence of Mn^2+^ and Mn^3+^ oxidation states within the samples, signifying a mixed oxidation state, indicating the catalyst’s redox property. As evidenced from Table S2, the Mn^2+^/Mn^3+^ ratio calculated for samples RCOP_CoMn_2_O_4_, COP_C_ CoMn_2_O_4_ and COP_M_ CoMn_2_O_4_ are 1.80, 1.77 and 1.60, respectively. This high Mn^2+^ content is significant as it facilitates the formation of oxygen vacancies to maintain charge neutrality, enhancing oxygen mobility and contributing to oxygen vacancies^33,34^. These features are critical for the catalyst’s redox properties and ability to activate oxygen during the soot oxidation. The mixed oxidation of states of Mn also promotes redox cycling, enabling efficient electron transfer and oxygen activation, which are essential for soot oxidation. Similarly, for the Co 2p spectra, two peaks around 780 eV and 795 eV correspond to Co 2p_3/2_ and Co 2p_1/2_, respectively, with a satellite peak around 786 eV. All the samples showed a spin-orbit energy of ~ 15.3 eV for all the samples. The peaks were further deconvoluted into ~ 780.28 eV and ~ 795.54 eV attributes to Co^2+,^ whereas ~ 782.23 eV and 797.03 eV attributes to Co^3+^. The peaks were in good accord with the literature^35,36^. The ratio of Co^2+^/Co^3+^ was found to be 2.23, 1.86 and 1.49 for the samples RCOP_CoMn_2_O_4_, COP_C_ CoMn_2_O_4_ and COP_M_ CoMn_2_O_4_, respectively (**Table S3**). The sample RCOP_CoMn_2_O_4_ exhibited a high content of low oxidation state of cobalt, which results in a high concentration of oxygen vacancies^37^. The coexistence of Co^2+^/Co^3+^ and Mn^2+^/Mn^3+^ redox pairs in CoMn_2_O_4_ enhances its redox properties. This combination allows for efficient electron transfer processes vital for soot oxidation^38,39^. For the O 1s peaks, three peaks with binding energy were observed: ~529 eV, 531 eV, and 532 eV. These peaks were ascribed to surface lattice oxygen species (O_Ι_), surface oxygen species(O_II_), and chemisorbed oxygen species (O_III_), respectively. In a typical spinel oxide, the intrinsic defects are found without any dopants, and these defects (Schottky defect) tend to form oxygen vacancies along with cation vacancies^40^. The concentration of these oxygen vacancies can be studied from the O 1s peaks. The concentration of oxygen vacancies in the synthesised samples is 20%, 19%and 18% for RCOP_ CoMn_2_O_4_, COP_ C_CoMn_2_O_4_ and COP_ M_CoMn_2_O_4,_ respectively. The concentration of oxygen vacancies is related to the active centres of the catalyst^15^.

Generally, the release of lattice oxygen species is associated with the redox properties of the metal ions on the catalyst surface. In contrast, the release of chemisorbed oxygen species is linked to the concentration of oxygen vacancies. A higher amount of chemisorbed oxygen species is required for a soot oxidation reaction to occur at a lower temperature^41^. Therefore, the active oxygen species estimated from XPS analysis are tabulated in **Table S4.** As shown in **Table S4**, the number of active oxygen species of the samples RCOP_ CoMn_2_O_4_, COP_ C_CoMn_2_O_4_ and COP_ M_CoMn_2_O_4_ is 0.36, 0.28 and 0.27, respectively. The calculated number of active oxygen species (O_II_, O_III_) can be related to the percentage of concentrated oxygen vacancies. Furthermore, a higher content of active oxygen species signifies enhanced surface catalytic properties^42^. Thus, an increased redox potential and active oxygen species in the sample RCOP_ CoMn_2_O_4_ signifies the formation of oxygen vacancies. The oxygen species are associated with the efficiency of catalyst activity. Low oxygen coordination and vacancies on the surface are necessary to achieve oxygen mobility and further facile oxygen transfer on the catalyst surface. Therefore, RCOP_ CoMn_2_O_4_ is likely to exhibit the highest activity for soot oxidation among the synthesised samples.

### Catalytic activity of soot oxidation

**Fig. 4:**
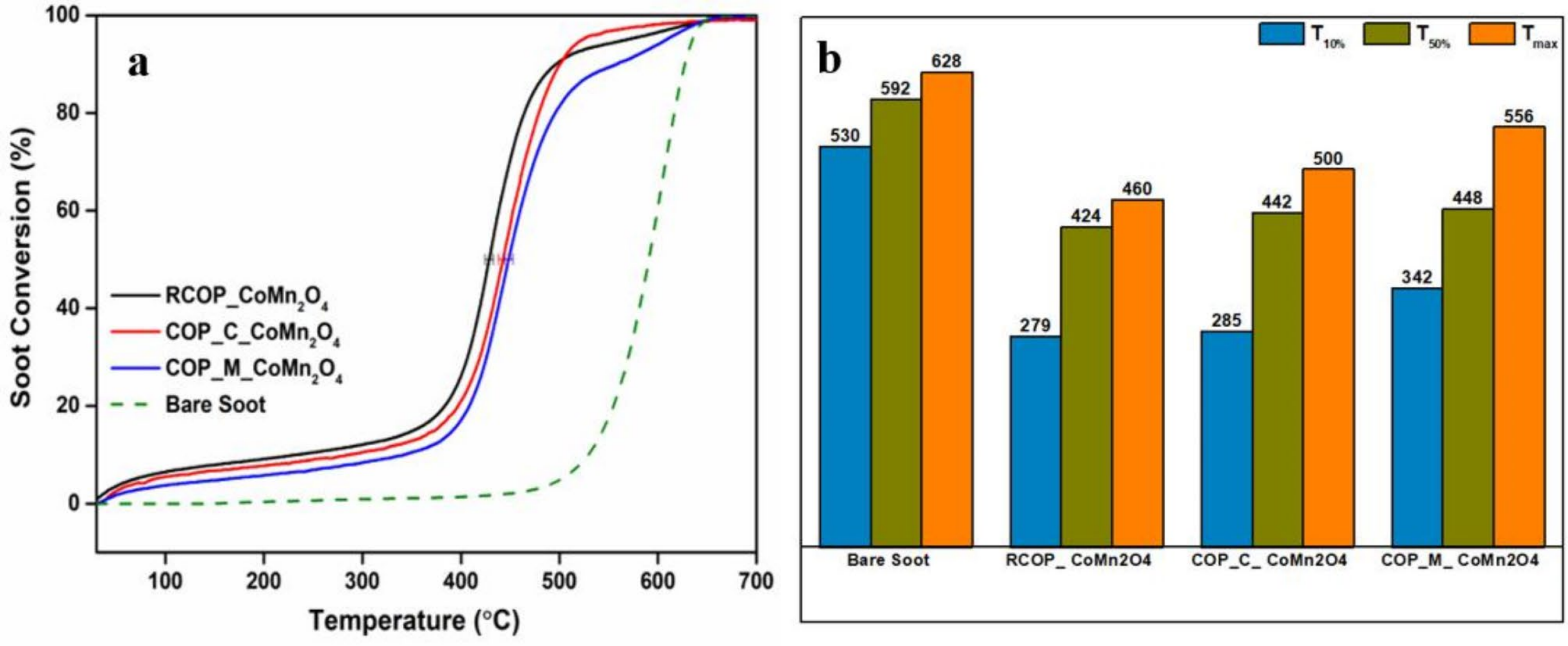
(**a**) Soot conversion profile of the catalysed and bare soot (**b**) Soot conversion temperatures of catalysts and bare soot.

The soot conversion profile of all synthesised catalysts, as depicted in Fig. 4**(a)**, highlights the effectiveness of RCOP_ CoMn_2_O_4_, COP_C_ CoMn_2_O_4_, and COP_M_ CoMn_2_O_4_ in facilitating soot oxidation. Figure 4**(b)** depicts the soot conversion temperatures for synthesised samples and compares its performance in **Table S5** with spinel oxides reported in the literature for soot oxidation activity. The T_50%_ values, representing the temperature at which 50% of the soot is converted, are 424 ± 4 °C, 442 ± 5 °C, and 448 ± 4 °C for RCOP_ CoMn_2_O_4_, COP_C_ CoMn_2_O_4_, and COP_M_ CoMn_2_O_4_, respectively. All catalysts initiate ignition around 300 °C, whereas uncatalysed soot ignites at approximately 530 °C. Compared to the bare soot, the T_50%_ of samples RCOP_ CoMn_2_O_4_, COP_C_ CoMn_2_O_4_, and COP_M_ CoMn_2_O_4_ are reduced by 168 °C, 150 °C, and 144 °C, respectively, indicating enhanced catalytic properties in the order: uncatalysed soot < COP_M_ CoMn_2_O_4_ < COP_C_ CoMn_2_O_4_ < RCOP_ CoMn_2_O_4_. These findings align with the soot temperature-programmed reduction (TPR) and XPS analysis, emphasising the oxygen species and metal ion’s redox nature.

The redox property of Co^3+/2+^ and Mn^2+/3+^ contributes to the soot oxidation by facilitating easy diffusion of oxygen ions from the catalyst lattice structure. This diffusion enhances the migration of oxygen ions to the catalyst’s surface when the surface-adsorbed oxygen species are depleted^43^. However, the migration of oxygen ions from the lattice structure is relatively slow, which results in higher soot oxidation temperatures. The same phenomenon is observed with the COP_M_ CoMn_2_O_4_ sample per soot TPR analysis. As discussed earlier in XPS and Raman results, the redox property and the presence of oxygen vacancies together contribute to the soot oxidation reaction. The catalytic soot oxidation can be further explained by relating the Raman and XPS results. The Raman spectra revealed distinct vibrational modes and peaks characteristic of CoMn_2_O_4_, including those indicative of oxygen vacancies, as evidenced by the blue shift around 650 cm⁻¹. These vacancies are critical in enhancing oxygen mobility and providing additional active sites for oxidation reactions. The XPS analysis harmonises the soot oxidation results. The active oxygen species was highest for the RCOP_CoMn_2_O_4_ sample, and lattice oxygen resulted in lower soot oxidation temperature.

### Soot oxidation kinetics of CoMn_2_O_4_ spinel

#### FWO and KAS methods to determine activation energy

**Fig. 5:**
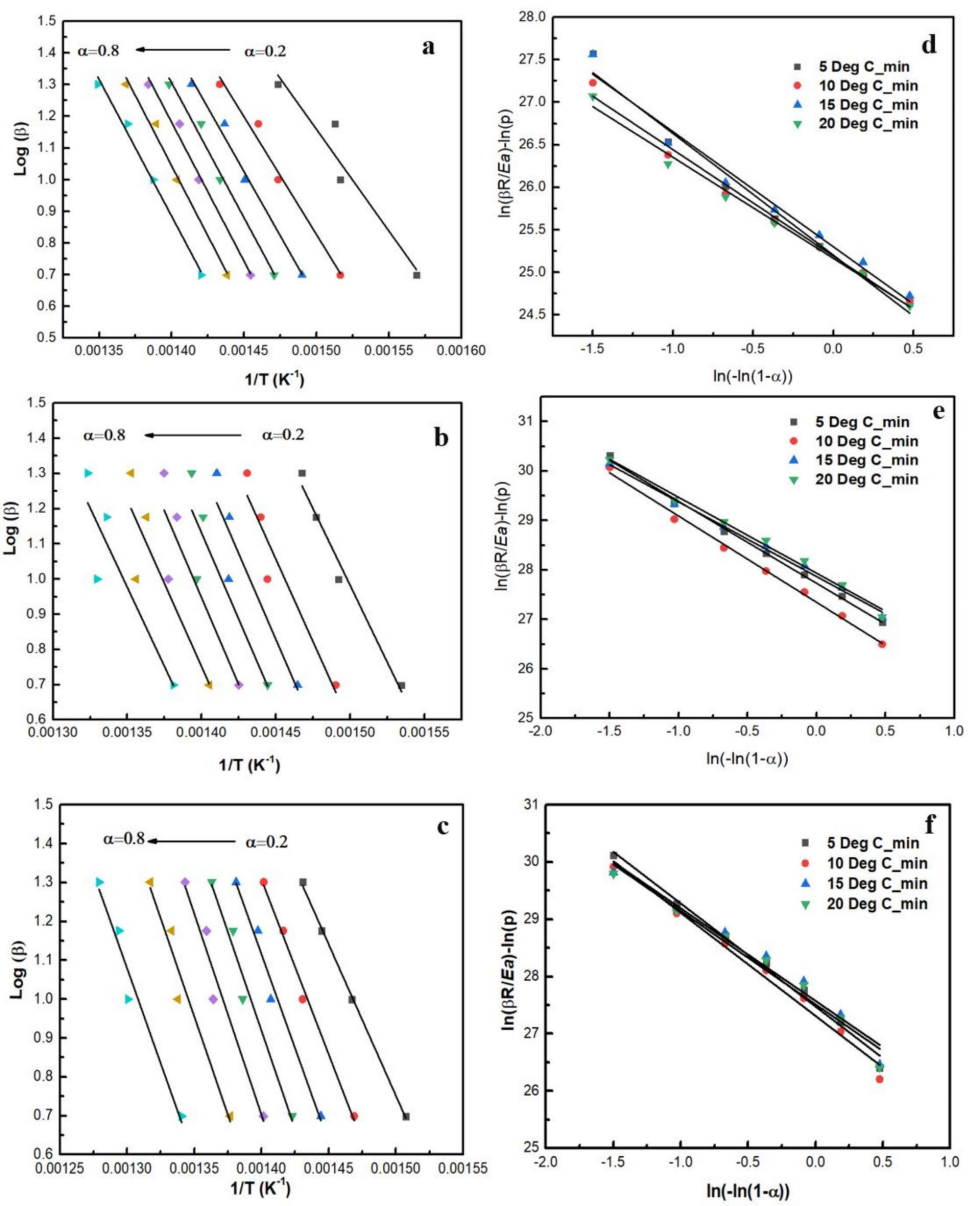

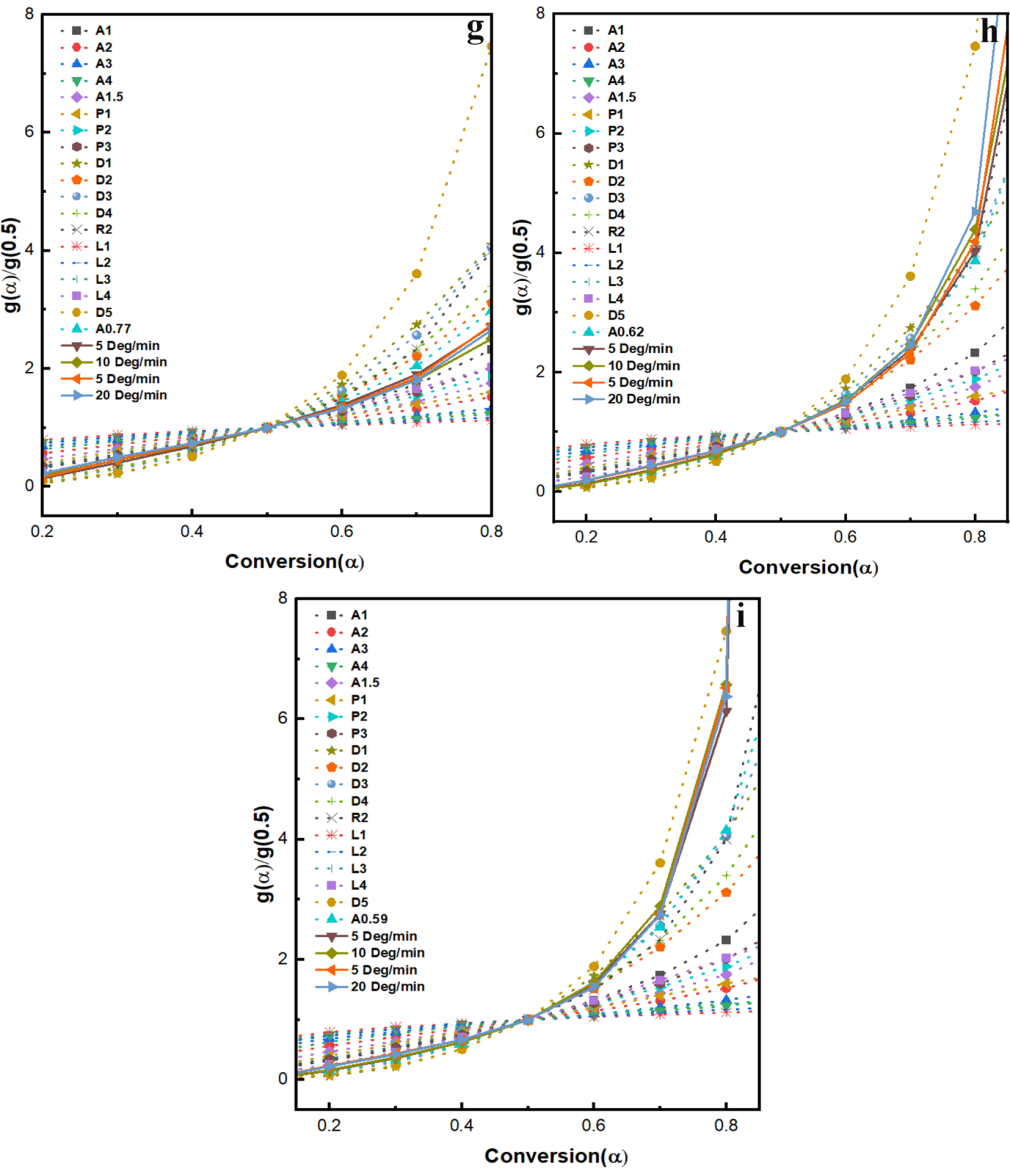
Ozawa plots of (**a**) RCOP_ CoMn_2_O_4_ (**b**) COP_C_ CoMn_2_O_4_, (**c**) COP_M_ CoMn_2_O_4_; Avrami-Erofeev plots of (**d**) RCOP_ CoMn_2_O_4_ (**e**) COP_C_ CoMn_2_O_4_, (**f**) COP_M_ CoMn_2_O_4_; Master Plots of theoretical and experimental data of (**g**) RCOP_ CoMn_2_O_4_ (**h**) COP_C_ CoMn_2_O_4_, (**i**) COP_M_ CoMn_2_O_4_.

The Flynn-Wall-Ozawa (FWO) and Kissinger-Akahira-Sunose (KAS) methods are among the most reliable techniques for determining the activation energy (Ea) of chemical reactions. These iso conversional methods involve measuring the temperature (T) at various heating rates (β) while maintaining a constant conversion (α). Figure 5a, b and c and S2 illustrate the application of these methods to the synthesised catalysts. Figure 3a and b, and 3c present the plot of log(β) against 1/T for each catalyst using the FWO approach. The slope of the resulting lines (-Ea/R) allows for calculating the activation energy. Similarly, Figure S2 shows the plot of ln(β/T²) against 1/T for each catalyst using the KAS method, where the slope again provides the activation energy values. The average activation energies calculated using these methods are summarised in Table S6, ranging from 153 kJ/mol to 173 kJ/mol. Yang et al.,^44^ reported the activation energy (136 kJ/mol) calculated through the Ozawa method of NiCo_2_O_4_ synthesised via the hydrothermal method. Lee et al.,^45^ synthesised CeO_2_ and Ag-doped CeO_2_ nanofibers and reported their activation energy of 123.2 kJ/mol and 112 kJ/mol, respectively. Yang et al. reported that the activation energy of CeO_2_ synthesised via the reflux method was 166.6 kJ/mol. It can be perceived that the preparation methods affect the activation energy of reactions. In this study, the RCOP_ CoMn_2_O_4_ sample exhibited the lowest activation energy at 153 kJ/mol, while the COP_M_ CoMn_2_O_4_ sample had the highest activation energy at 173 kJ/mol. In comparison, non-catalytic soot oxidation typically requires an activation energy of 211 kJ/mol (Su et al.). A decrease in activation energy is known to accelerate chemical reactions. The RCOP_CoMn_2_O_4_ sample, with an activation energy of 153 kJ/mol, significantly reduces compared to non-catalytic soot oxidation, suggesting enhanced catalytic efficiency. According to the data, the FWO and KAS methods provided the lowest Ea values for the RCOP_CoMn_2_O_4_ sample, measured at 153.62 kJ/mol and 148.85 kJ/mol, respectively. For the spontaneous combustion of soot, activation energy generally falls between 150 and 170 kJ/mol, supporting the findings for the synthesised catalysts^46^. The findings show that the decrease in activation energy increases the reaction rate of the chemical process, which aligns with the observations of Ganiger et al.,^47^. The Ozawa method is considered more accurate than the KAS method; thus, the values obtained from the Ozawa method are used for further kinetic studies.

#### Determination of pre-exponential factor

Avrami–Erofeev (Am) approach derives non-integer exponent, m (Avrami exponent) and A (pre-exponential factor) using average activation energy from the Ozawa method. The best-fit model corresponds to the Am function, which indicates the complexity of the reaction kinetics. By plotting [ln(βR/E) – ln p(x)] against ln[-ln(1 – α)], as shown in Fig. 5d, e and f, the data demonstrates that the lines at different heating rates are nearly superimposed, indicating that the reaction model is independent of the heating rate^48^.

The calculated m and ln(A) values are presented in **Table S6**, with ln(A) ranging from 25 to 27 min⁻¹. From the literature, it is reported that the highest ln(A) value suggests a higher probability of soot particle collisions with the catalyst surface and, thus, shows a greater catalytic activity^49^. In this study, COP_C_ CoMn_2_O_4_ exhibited the highest ln(A) value of 27.72 min^− 1^ with an activation energy of 171.56 kJ/mol. However, RCOP_ CoMn_2_O_4_ exhibited superior catalytic activity with ln(A) 25.21 min^− 1^ and possessed low activation energy 153.62 kJ/mol. Triyono et al.,^50^ states that the overall catalytic activity is not determined by activation energy alone. The pre-exponential factor plays a crucial role. Even if the activation energy is high, a sufficiently large pre-exponential factor can maintain or even enhance the catalyst’s activity, balancing the negative impact of the high energy barrier. From the literature, Nascimento et al.,^51^ reported that the ln (A) value of CeO_2_ was 19.73 min^− 1^, and the ln (A) value increased to 27.73 min^− 1^ with doping of zinc ions. Zouaoui et al.,^52^ noticed that the ln (A) value of CeO_2_ increased from 5.8 to 6.2 min^− 1^ when the reactions were carried out in the O_2_ and NO atmosphere.

#### Master plots

Figure 5g and h, and 5i display the master plots for RCOP_ CoMn_2_O_4_, COP_C_ CoMn_2_O_4_, and COP_M_ CoMn_2_O_4_ samples corresponding to the g(α) functions as reported in López-Fonseca et al.^48^. Comparing theoretical and experimental master plots allows the identification of the reaction model, which is crucial for understanding solid-gas reactions^53^. If the reaction follows A1, nucleation and growth process 41 are the rate-determining steps. In the Power law model (L1 to L4), the nucleation rate is set to follow power law, and nuclei growth is assumed constant. The rate equation depends upon the shape factor in diffusion mode (D1 to D3)^54^. The Ginstling-Brounshtein model applies to diffusion-controlled processes^55^. The conversion models for the samples differ: RCOP_ CoMn_2_O_4_ follows a combination of nucleation and growth model (A1), power law (L4), second-order reaction (R2), 2-dimensional diffusion model (D2), and Ginstling-Brounshtein model (D4). In contrast, COP_C_ CoMn_2_O_4_ and COP_M_ CoMn_2_O_4_ follow the power law (L4), second-order reaction (R2), 2-dimensional diffusion model (D2), and Ginstling-Brounshtein model (D4). The behaviour and efficiency of the catalysts in oxidising soot are influenced by the specific dopants or support materials used and the extent of soot conversion. This highlights the importance of material selection and its impact on catalytic performance and soot oxidation mechanisms.

Patil et al.,^56^ have reported no direct correlation between activation energy, pre-exponential factor, and T_50%_ temperature, underscoring the complexity of these reactions. The insights gained from these analyses are critical for optimising catalyst design and improving the efficiency of soot oxidation processes.

#### Experimental and theoretical curves construction and comparison

**Fig. 6:**
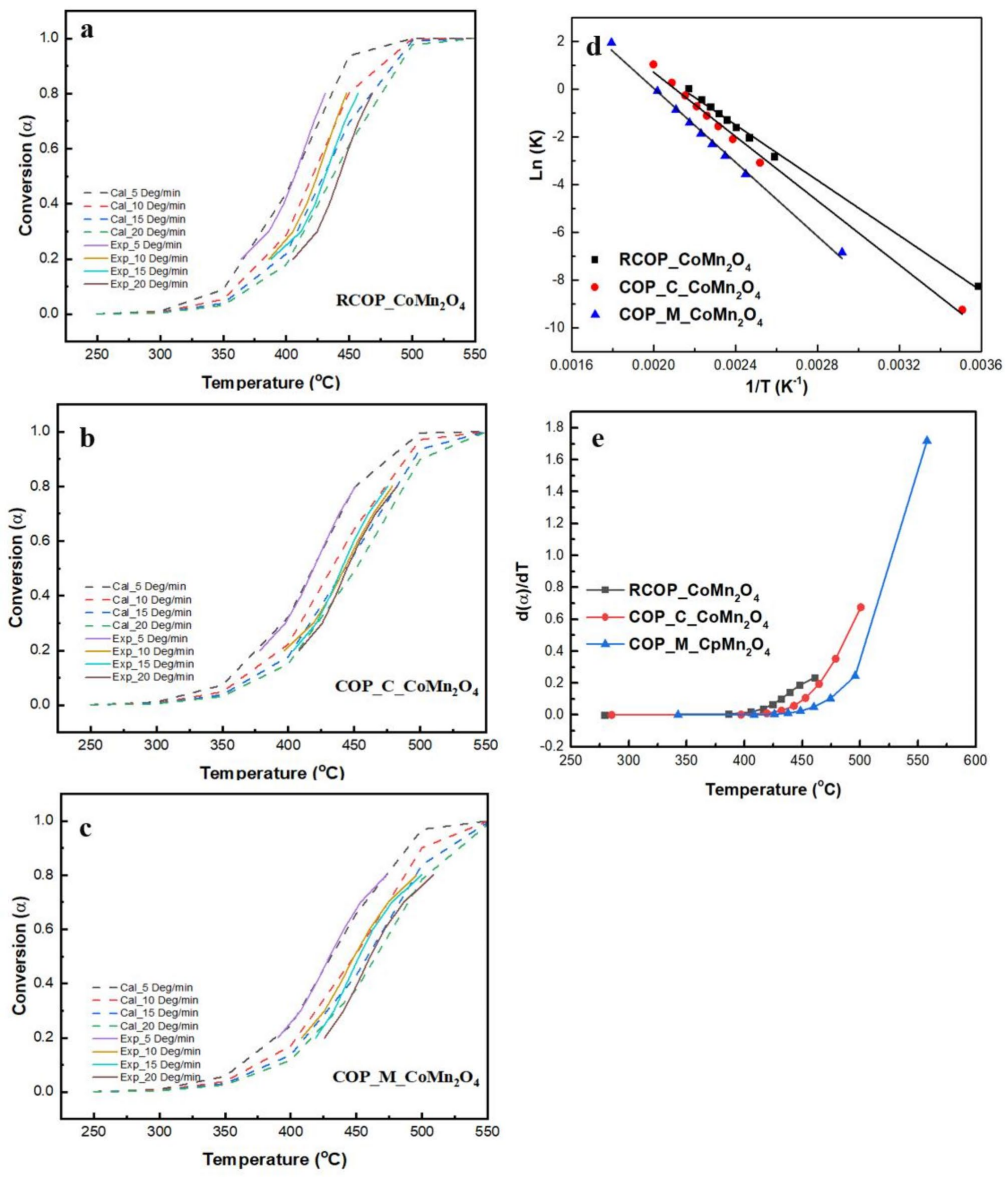
Experimental and calculated thermoanalytical curves of (**a**) RCOP_ CoMn_2_O_4,_ (**b**) COP_C_ CoMn_2_O_4_, (**c**) COP_M_ CoMn_2_O_4_; (**d**) Rate vs. temperature at a heating rate of 10 °C/min (**e**) Arrhenius plot at a heating rate of 10 °C/min.

Figure 6a and b, and 6c display thermoanalytical curves constructed from experimental and computed data for each heating rate. The prepared sample’s experimental data strongly correspond to the predicted curves regardless of heating rates, indicating that the proposed kinetic analysis was appropriate for modelling soot combustion.

In mathematics, the rate of solid-state reactions can be described using the differential kinetic reaction model (f(α)) and the rate constant (k(T))^48^. The reaction rate can be calculated using previously determined kinetic parameters, as illustrated in Fig. 6d. It is observed that as the conversion (α) increases, the reaction rate also increases. This trend is observed in the case of the COP_M_ CoMn_2_O_4_ catalyst, with its reaction rate much higher than those of RCOP_CoMn_2_O_4_ and COP_C_ CoMn_2_O_4_, as seen in Fig. 6d.

Further, the kinetic analysis can be analysed using the slope of the Arrhenius plot ((ln(k)) versus (1/T)). The Arrhenius equation explains the rate constant of the reaction as related to temperature, thus explaining the reaction’s activation energy and frequency factor. The kinetic activity of the catalysts decreases from RCOP_CoMn_2_O_4_ to COP_M_ CoMn_2_O_4_, as witnessed in Fig. 6e.

The higher reaction rate and kinetic activity of RCOP_CoMn_2_O_4_ can be attributed to its superior catalytic properties. This may be due to better dispersion of active sites, higher surface area, or more favourable interactions between the catalyst and soot particles. These findings highlight the importance of catalyst design and optimisation in enhancing the efficiency of soot oxidation processes. The comparative analysis of the catalysts underscores the potential of RCOP_CoMn_2_O_4_ as a highly effective catalyst for soot combustion, offering valuable insights for developing advanced catalytic materials.

Characterisation techniques such as XRD, SEM, Raman, XPS and Soot TPR demonstrate that the synthesis of RCOP_CoMn_2_O_4_ via reverse co-precipitation leads to the formation of spherical particles. Herein, a significant shift of characteristic peak in Raman spectra confirms the presence of structural defects that have disturbed the vibrational frequency of oxygen atoms, which showcase the presence of oxygen vacancies. Similar behaviour was witnessed by Trung et al.,^57^ oxygen vacancies in Bi_2_WO_6_ cause a blue shift in the WO_6_ octahedral vibrations. Gao et al.,^58^ found that the blue shift A1 phonon mode of ZnO was attributed to point defects and oxygen vacancies. The synergistic effect of Mn^2+^/Mn^3+^ and Co^2+^/Co^3+^ facilitates efficient electron transfer and oxygen activation. The sample RCOP_CoMn_2_O_4_ exhibited the highest proportion of lower valence states, and hence, it tends to have a better catalytic activity due to enhanced redox property and oxygen vacancies^59,60^. Consequently, RCOP_CoMn_2_O_4_ exhibited better soot oxidation activity among the synthesised samples (T_50_ = 424 °C).

Kinetic triplets, which include the activation energy, pre-exponential factor, and reaction model, are crucial for understanding and optimising soot oxidation processes. These parameters help predict the soot oxidation rate under various conditions, which are essential for improving combustion efficiency and reducing emissions. Activation energy is a critical parameter that influences the temperature sensitivity of the soot oxidation reaction. The pre-exponential factor is another crucial kinetic parameter that affects the frequency of successful collisions leading to a reaction. The reaction model describes the mechanism of soot oxidation. Therefore, it is highly necessary to understand the catalytic efficiency and optimise the catalyst’s performance towards soot oxidation. In this study, RCOP_CoMn_2_O_4_ exhibited low activation energy 153.62 kJ/mol and low pre-exponential factor (25.21 min^− 1^) and followed a combination of nucleation and growth model (A1), power law (L4), second-order reaction (R2), 2-dimensional diffusion model (D2), and Ginstling-Brounshtein model (D4) among the synthesised samples.

The structural defects and oxygen vacancies identified through Raman and XPS analyses enhance oxygen activation and electron transfer, leading to low activation energy. The shift in the Raman spectrum, indicative of oxygen vacancies, suggests disrupted oxygen vibrational frequencies, which enhance oxygen mobility and activation, resulting in reduced activation energy. The soot TPR confirms the catalyst’s ability to release active oxygen species at lower temperatures, aligning with the low activation energy observed. The observed Mn^2+^/Mn^3+^ and Co^2+^ /Co^3+^ transitions (XPS data) enhance the redox properties, which directly support the catalytic efficiency indicated by the kinetic triplets. Additionally, the concentration of oxygen vacancies quantified from XPS data shows an inverse relationship with activation energy, as higher vacancy concentrations facilitate oxygen activation, lowering the energy barrier for soot oxidation. The activation energy of soot oxidation is directly related to T_50%_; higher activation energies result in higher T_50%_ values, indicating that more energy is required for oxidation, thus reducing soot reactivity^61,62^. The sample RCOP_CoMn_2_O_4_ exhibited low T_50%_ (424 °C) and low activation energy among the synthesised samples. Further, the high redox activity of Mn and Co species promotes efficient catalytic behaviour, as reflected in the low pre-exponential factor, emphasising the synergy between characterisation and kinetic performance. The low pre-exponential factor reflects the reaction’s reliance on structural and electronic properties, such as the availability of oxygen vacancies and efficient redox activity, to enhance catalytic performance. These findings demonstrate the synergy between characterisation results and kinetic performance, providing a comprehensive understanding of the catalyst’s behaviour.

## Conclusion

The following conclusions were drawn from this study.

1. RCOP_ CoMn_2_O_4,_ COP_C_ CoMn_2_O_4_, and COP_M_ CoMn_2_O_4_ samples were synthesised via reverse and co-precipitation methods. All the samples displayed a tetragonal structure.
2. Raman spectra analysis confirmed the presence of lattice oxygen in RCOP_ CoMn_2_O_4_ and oxygen vacancies in all synthesised samples.
3. XPS analysis showed the co-existence of Co^3+/2+^ and Mn^2+/3+^ in synthesised samples, which contributes to the formation of oxygen vacancies due to their continuous redox reaction within the lattice structure.
4. The soot TPR profile revealed the presence of two types of oxygen species essential for soot oxidation in RCOP_ CoMn_2_O_4_ and COP_C_ CoMn_2_O_4_ samples. On the other hand, COP_M_ CoMn_2_O_4_ displayed only lattice oxygen species.
5. COP_C_ CoMn_2_O_4_ displayed the lowest T_50%_ (430 °C) amongst other catalysts. The better catalytic activity can be attributed to the catalyst’s high active oxygen species and redox properties.
6. The kinetic triplets were evaluated for all synthesised samples. RCOP_ CoMn_2_O_4_ displayed the lowest activation energy, and COP_C_ CoMn_2_O_4_ displayed the highest pre-exponential factor. The activity and rate of reaction are high for RCOP_ CoMn_2_O_4_.

## Electronic supplementary material

Below is the link to the electronic supplementary material.

Supplementary Material 1

Supplementary Material 2

## Data Availability

The datasets used and/or analysed during the current study available from the corresponding author on reasonable request.
