# Supplementary material for: Potential of CoMn2O4 spinel as soot oxidation catalyst and its kinetics thereof
Source: Sci Rep. 2025 Jan 7;15:1174. doi: 10.1038/s41598-025-85736-2 (PMC11706943; doi:10.1038/s41598-025-85736-2)
Supplement: Supplementary file 1 — Supplementary Material 1 [file 41598_2025_85736_MOESM1_ESM.docx]

**Potential of CoMn_2_O_4_ Spinel as Soot Oxidation Catalyst and its Kinetics thereof**

*Nithya R ^a^, Sunaina S Patil ^b^, Hari Prasad Dasari ^b^, Harshini Dasari ^a^, Nethaji S ^a^*

*^a^ Department of Chemical Engineering, Manipal Institute of Technology,*

*Manipal Academy of Higher Education, Manipal 576104, India*

***^b^*** *Energy & Catalysis Materials Laboratory, Department of Chemical Engineering,*

*National Institute of Technology Karnataka, Surathkal, Mangalore 575025*

**Equations**

$$d= \frac{k \lambda}{\beta cos \theta} eq (S1)$$

Where, d is average crystallite size, k is the shape factor, λ is the X-ray wavelength, β is the broadening at half of the maximum intensity, and θ is the incident angle.

1. Ozawa method's mathematical expression

$\log\left( ß \right)+0.4567\frac{Ea}{RT}=constant$ eq (S2)

1. KAS method's mathematical expression

$ln\frac{\boldsymbol{ß}}{T^{2}}=ln \left[ \frac{AEa}{Rg(\alpha)} \right]- \frac{Ea}{RT}$ eq (S3)

1. Master plot for non-isothermal conditions

$\frac{g(\alpha)}{g(0.5)}= \frac{p(x)}{p(0.5)}$ eq (S4)

$g(\alpha)= \frac{AEa}{ßR} p(x)$ eq (S5)

1. Coats – Redfern Method is represented by below equation

$ln\frac{\boldsymbol{g(\alpha)}}{T^{2}}=\ln\left( \frac{AR}{\boldsymbol{ßEa}}\left[ 1-\frac{2RT}{Ea} \right] \right)- \frac{Ea}{RT}$ eq (S6)

1. Determination of Pre -exponential factor

$ln\frac{ßR}{Ea}-\ln p\left( x \right) =\ln A-\frac{1}{m}ln\left[ -\ln(1-\alpha) \right]$ eq (S7)

**Morphological analysis**

The formation of the RCOP_ CoMn_2_O_4_ sample through reverse co-precipitation involves the precursor ions forming a complex with the precipitation agent. The cobalt ions and manganese ions are added dropwise to the ammonia solution, suggesting the controlled nucleation and growth of nanoparticles. Two complexes Mn (H_2_O)_4_(OH)_2_ and Co (NH_3_)_5_(H_2_O)^3+^ formed during the contact of ammonia solution helps to control the nucleation and growth of the nanoparticles ^12^. The complex formation reduces the concentration of the precursor ions in the solution, preventing rapid and uncontrolled nucleation. During the calcination process, the phase CoMn_2_O_4_ is formed. The formation of COP_ C_CoMn_2_O_4_ involves solution-based oxidation and precipitation of manganese and cobalt ions in ammonia solution, followed by crystallization during the calcination process. Before dropping the cobalt ionic solution into the ammonia solution, the Co^2+^ ions get easily oxidized into Co^3+^ ions. The Co^3+^ ions, once in contact with the ammonia solution produces Co (NH_3_)_4_^3+^ and the subsequent addition of manganese ions produces MnOOH. Due to the high redox property of Co (NH_3_)_4_^3+^ further oxidizes MnOOH to intermediate Mn_7_O_13_ ^3^. The formation of COP_ C_CoMn_2_O_4_ involves solution-based precipitation of manganese and cobalt ions in an ammonia solution. When ammonia solution is dropped into manganese solution, the manganese ions precipitate into MnOOH. The Co^3+^ ions, once in contact with the ammonia solution, produces Co (NH_3_)_4_^3+,^ and during the calcination process, the phase of CoMn_2_O_4_ is formed (Wang et al 2017).

**Fig S1.** shows the SEM images of RCOP_ CoMn_2_O_4_, COP_C_ CoMn_2_O_4_, and COP_M_ CoMn_2_O_4_ samples. From the SEM images, it is observed that the synthesized samples had seed-like morphology with rough surfaces. The presence of surface roughness in all samples may act as an active site for soot; it enhances the efficiency of soot oxidation. RCOP_ CoMn_2_O_4_ sample has high surface roughness and finer seed-like morphology than other samples. The formation of finer nanoparticles can be attributed to the controlled nucleation formation during the reaction time. Meanwhile, COP_M_ CoMn2O4 samples exhibited smooth and well-defined seed-like morphology, and the particle size seemed to have increased compared to other samples. Accordingly, the BET surface area of RCOP_ CoMn_2_O_4_, COP_C_ CoMn_2_O_4_, and COP_M_ CoMn_2_O_4_ follows the same trend as seen in SEM, RCOP_ CoMn_2_O_4_ >COP_C_ CoMn_2_O_4_, > COP_M_ CoMn_2_O_4_.

**Table S1.** Unit cell parameters and BET of synthesised samples.

| **Sample Name** | **a=b(Å)** | **c(Å)** | **Phase** | **Volume (Å^3^)** | **D (nm)** | **Surface Area (m^2^/g)** |
| --- | --- | --- | --- | --- | --- | --- |
| RCOP_ CoMn_2_O_4_ | 8.13 | 9.32 | Tetragonal | 617.31 | 22.97 | 10.88 |
| COP_C_ CoMn_2_O_4_ | 8.15 | 9.34 |  | 622.03 | 21.13 | 7.34 |
| COP_M_ CoMn_2_O_4_ | 8.09 | 9.33 |  | 618.18 | 24.63 | 6.34 |

| **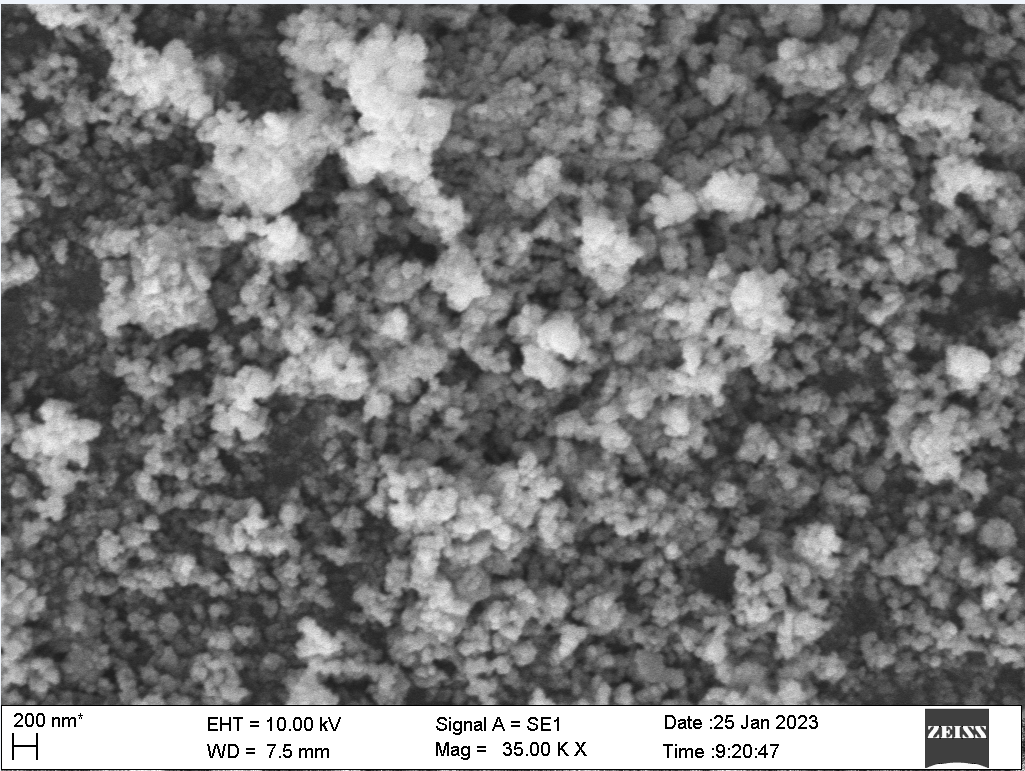** | **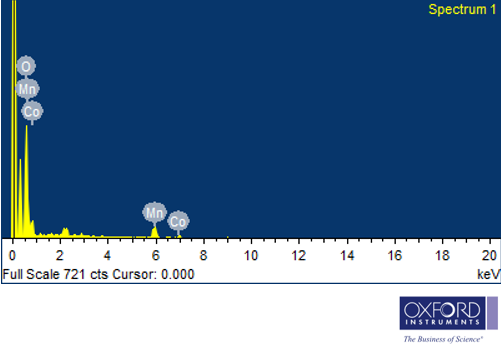** |
| --- | --- |
| **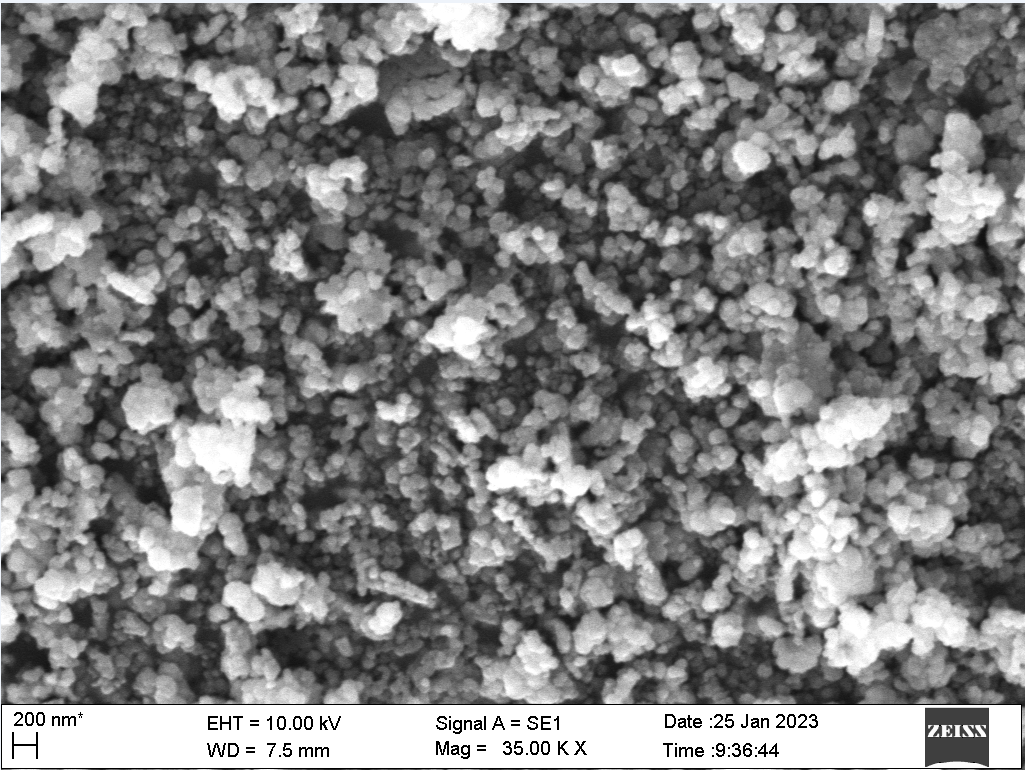**  **(b)** | **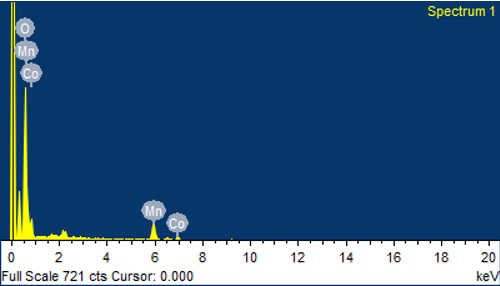** |
| **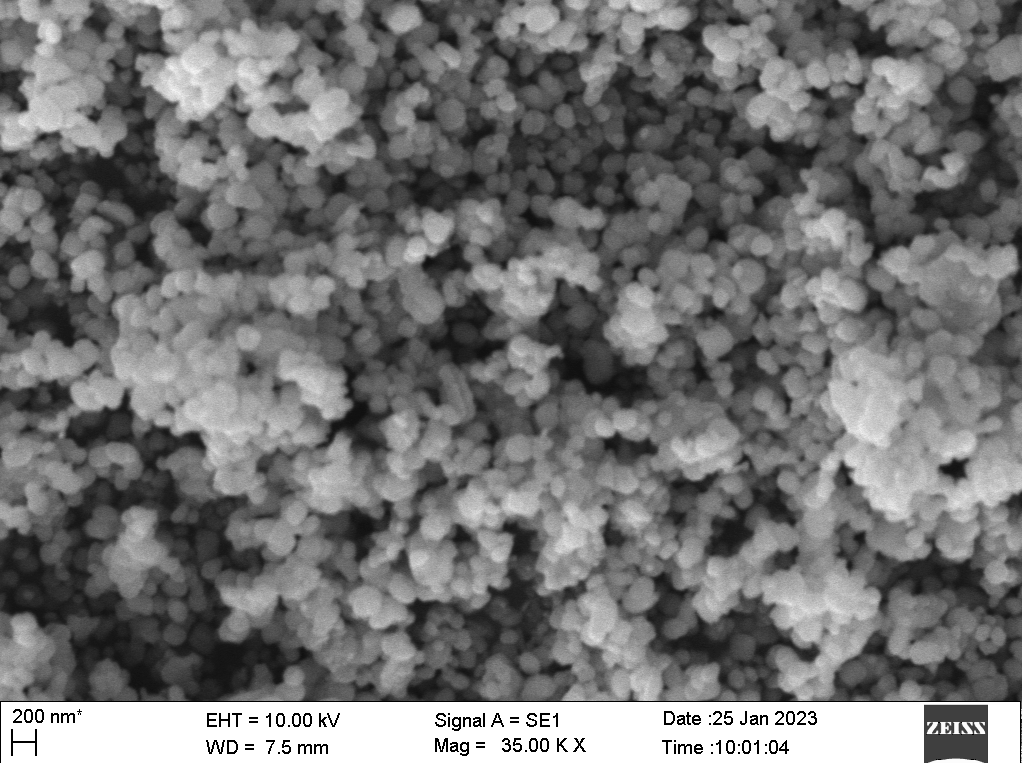**  **(c)** | **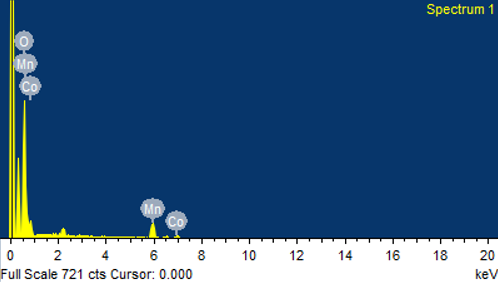** |

**(a)**

**Fig S1.** SEM and EDX images of (a) RCOP_ CoMn_2_O_4_ (b) COP_C_ CoMn_2_O_4_, (c) COP_M_ CoMn_2_O_4_

| **Sample Name** | **Element** | **B.E. (eV)** | **Area** | **Active Oxygen Species** | **Concentration of Oxygen Vacancy (%)** |
| --- | --- | --- | --- | --- | --- |
| RCOP_ CoMn_2_O_4_ | O_I_ | 529.982 | 31150.360 | 0.36 | 20 |
|  | O_II_ | 531.544 | 37671.730 |  |  |
|  | O_III_ | 532.907 | 121992.100 |  |  |
| COP_C_ CoMn_2_O_4_ | O_I_ | 529.710 | 135304.500 | 0.28 | 19 |
|  | O_II_ | 530.806 | 35117.310 |  |  |
|  | O_III_ | 531.693 | 17636.120 |  |  |
| COP_M_ CoMn_2_O_4_ | O_I_ | 529.929 | 187569.700 | 0.27 | 18 |
|  | O_II_ | 531.249 | 45738.540 |  |  |
|  | O_III_ | 532.541 | 27025.210 |  |  |

**Table S2**. Estimation of active oxygen species from XPS analysis

**Table S3**. T_50%_ °C of various spinel oxides for soot oxidation activity

| **Sl.No.** | **Spinel Oxide** | **T_50%_ °C** | **Reference** |
| --- | --- | --- | --- |
| 1 | CuFe_2_O_4_ | 488 | ^4^ |
| 2 | MnCo_2_O_4_ | 504 | ^5^ |
| 3 | NiCo_2_O_4_ | 585 | ^6^ |
| 4 | CuCo_2_O_4_ | 574 | ^6^ |
| 5 | ZnCo_2_O_4_ | 569 | ^6^ |
| 6 | Co_3_O_4_ | 580 | ^6^ |
| 7 | ZnAl_2_O_4_ | 630 | ^7^ |
| 8 | CuCr_2_O_4_ | 571 | ^8^ |
| 9 | CuMn_2_O_4_ | 565 | ^8^ |
| 10 | CuFe_2_O_4_ | 489 | ^9^ |
| 11 | CoFe_2_O_4_ | 600 | ^9^ |
| 12 | NiFe_2_O_4_ | 543 | ^9^ |

**Table S4**. Kinetic parameters of synthesized samples

| **Sample Code** | **Avg.Activation Energy** | | **Avg.Pre- Exponential Factor (Ln(A))** | **Avrami Integer** | **Reaction Model** |
| --- | --- | --- | --- | --- | --- |
|  | **Ea (Ozawa)** | **Ea (KAS)** | **Am** |  |  |
|  | **kJ/mol** | | **min^-1^** | **m** |  |
| RCOP_ CoMn_2_O_4_ | 153.62 | 148.85 | 25.21 | 0.77 | A1, L4, R2, D2 and D4 |
| COP_C_ CoMn_2_O_4_ | 171.56 | 170.82 | 27.72 | 0.62 | L4, R2, D2 and D4 |
| COP_M_ CoMn_2_O_4_ | 173.72 | 170.35 | 27.46 | 0.58 | L4, R2, D2 and D4 |

**
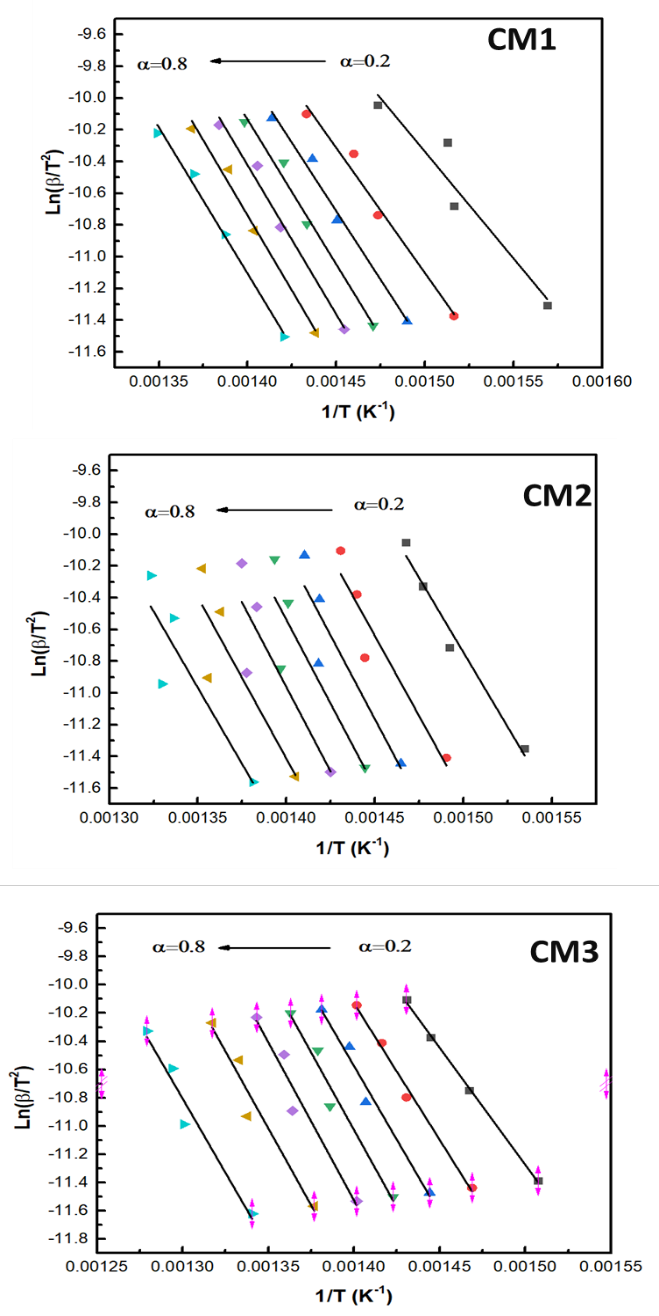
**

**Figure S2.** KAS method plots

**References**

1. Moore, T. ., Ellis, M. & Selwood, P. . T. e. moore, marylinn ellis. *J. Am. Soc.* **72**, 856–866 (1950).

2. Li, Y., Tan, B. & Wu, Y. Freestanding mesoporous quasi-single-crystalline Co3O 4 nanowire arrays. *J. Am. Chem. Soc.* **128**, 14258–14259 (2006).

3. Shashidharagowda, H., Mathad, S. N. & Abbigeri, M. B. Structural, Vibrational and Magnetic Characterization of Copper doped CoMn2O4 Nano-particles Synthesized by Chemical Route. *Sci. Sinter.* **53**, 429–444 (2021).

4. Li, T. *et al.* Investigation of alkali metals addition on the catalytic activity of CuFe2O4 for soot oxidation. *Sep. Purif. Technol.* **283**, 120224 (2022).

5. Xu, K., Zhang, Y., Shan, W. & He, H. Promotional Effects of Sm/Ce/La Doping on Soot Oxidation over MnCo2O4Spinel Catalysts. *J. Phys. Chem. C* **125**, 26484–26491 (2021).

6. Zhang, S. *et al.* Study on catalytic soot oxidation over spinel type ACo2O4 (A = Co, Ni, Cu, Zn) catalysts. *Aerosol Air Qual. Res.* **17**, 2317–2327 (2017).

7. Zawadzki, M., Staszak, W., López-Suárez, F. E., Illán-Gómez, M. J. & Bueno-López, A. Preparation, characterisation and catalytic performance for soot oxidation of copper-containing ZnAl2O4 spinels. *Appl. Catal. A Gen.* **371**, 92–98 (2009).

8. ChemistrySelect - 2021 - Zhang - Activity and Stability of Cu‐Based Spinel‐Type Complex Oxides for Diesel Soot Combustion.pdf.

9. Chen, H., Li, T., Xu, Z., Wang, W. & Wang, H. Oxidation of soot promoted by Fe-based spinel catalysts. *Mater. Res. Express* **9**, (2022).
